# Nomenclature of Genetically Determined Myoclonus Syndromes: Recommendations of the International Parkinson and Movement Disorder Society Task Force

**DOI:** 10.1002/mds.27828

**Published:** 2019-10-04

**Authors:** Sterre van der Veen, Rodi Zutt, Christine Klein, Connie Marras, Samuel F. Berkovic, John N. Caviness, Hiroshi Shibasaki, Tom J. de Koning, Marina A.J. Tijssen

**Affiliations:** ^1^ Department of Neurology University Groningen, University Medical Center Groningen Groningen Netherlands; ^2^ Department of Neurology Haga Teaching Hospital The Hague The Netherlands; ^3^ Institute of Neurogenetics University of Lübeck Lübeck Germany; ^4^ Edmond J. Safra Program in Parkinson's Disease Toronto Western Hospital, University of Toronto Toronto Ontario Canada; ^5^ Epilepsy Research Center, Department of Medicine University of Melbourne, Austin Health Heidelberg Victoria Australia; ^6^ Department of Neurology Mayo Clinic Scottsdale Arizona USA; ^7^ Kyoto University Graduate School of Medicine Kyoto Japan; ^8^ Department of Genetics University of Groningen, University Medical Centre Groningen Groningen The Netherlands

**Keywords:** genetics, hyperekplexia, myoclonic epilepsy, myoclonus, nomenclature

## Abstract

Genetically determined myoclonus disorders are a result of a large number of genes. They have wide clinical variation and no systematic nomenclature. With next‐generation sequencing, genetic diagnostics require stringent criteria to associate genes and phenotype. To improve (future) classification and recognition of genetically determined movement disorders, the Movement Disorder Society Task Force for Nomenclature of Genetic Movement Disorders (2012) advocates and renews the naming system of locus symbols. Here, we propose a nomenclature for myoclonus syndromes and related disorders with myoclonic jerks (hyperekplexia and myoclonic epileptic encephalopathies) to guide clinicians in their diagnostic approach to patients with these disorders. Sixty‐seven genes were included in the nomenclature. They were divided into 3 subgroups: prominent myoclonus syndromes, 35 genes; prominent myoclonus syndromes combined with another prominent movement disorder, 9 genes; disorders that present usually with other phenotypes but can manifest as a prominent myoclonus syndrome, 23 genes. An additional movement disorder is seen in nearly all myoclonus syndromes: ataxia (n = 41), ataxia and dystonia (n = 6), and dystonia (n = 5). However, no additional movement disorders were seen in related disorders. Cognitive decline and epilepsy are present in the vast majority. The anatomical origin of myoclonus is known in 64% of genetic disorders: cortical (n = 34), noncortical areas (n = 8), and both (n = 1). Cortical myoclonus is commonly seen in association with ataxia, and noncortical myoclonus is often seen with myoclonus‐dystonia. This new nomenclature of myoclonus will guide diagnostic testing and phenotype classification. © 2019 The Authors. *Movement Disorders* published by Wiley Periodicals, Inc. on behalf of International Parkinson and Movement Disorder Society.

Myoclonus is a hyperkinetic movement disorder characterized by sudden, brief, involuntary jerks of a single or multiple muscles.^1^, ^2^, ^3^ It can be caused by muscle contraction (positive myoclonus) or sudden interruption of muscle activity during intended isometric contraction (negative myoclonus).^4^ The myoclonic jerks can be difficult to distinguish from other hyperkinetic movement disorders.^5^ Electrophysiological testing has proven helpful for discriminating myoclonus from other hyperkinetic movement disorders and for classifying the myoclonus subtype.^6^ Myoclonus can be classified based on anatomical origin: cortical, subcortical (or noncortical^7^), spinal, and peripheral myoclonus.^6^ So far, in genetic myoclonus syndromes only cortical (CM) and subcortical subtypes have been described.^8^

Determination of the etiology of myoclonus is challenging, and recently, a novel diagnostic 8‐step algorithm was proposed to help clinicians accurately, efficiently, and cost‐effectively diagnose myoclonus.^8^ Once the acquired forms and late‐onset neurodegenerative disorders (such as Alzheimer's disease and parkinsonian disorders) of myoclonus are excluded in this diagnostic workup, a large number of genetically determined disorders with wide clinical variation remain. In almost all genetic syndromes, myoclonus is not the sole feature, but it is accompanied or even overshadowed by another movement disorder.^5^ This is likely the reason systematic nomenclature similar to PARK (for parkinsonism) or DYT (for dystonia) has not been established for myoclonus. In many of the suspected genetic myoclonus syndromes, the genetic cause is (still) unknown, but next‐generation sequencing (NGS) has revolutionized molecular genetic diagnosis and has produced an exponential increase in known genetic causes and expansion of movement disorder phenotypes, including myoclonus. However, NGS frequently produces genetic variants for which pathogenicity is unclear. This emphasizes the importance of good clinical phenotyping and weighting of NGS results in the context of the presenting clinical syndrome.

In 2012, the International Parkinson and Movement Disorder Society Task Force for Nomenclature of Genetic Movement Disorders was established to revise the system of locus symbols, as the current movement disorders system had become outdated with the advances in NGS, the lack of established criteria for conferring locus symbols, or ongoing revision of the list.^9^

Here we present a new myoclonus nomenclature. We also include groups of related disorders that can present in the outpatient clinic of a movement disorder specialist with *jerks* as a prominent symptom. First, there are the hyperekplexias, as the excessive startle reflex closely resembles reticular reflex myoclonus, both clinically and neurophysiologically.^10^ Second are the genetic epilepsy syndromes with myoclonic jerks, specifically the epileptic encephalopathies. Patients with myoclonic epilepsy encephalopathies exhibit, next to their clear epileptic attacks, often spontaneous, reflex or action myoclonus, with evidence of a cortical origin. These cortically driven epileptic jerks resemble isolated cortical myoclonus, as both are characterized by short‐lasting (<100‐millisecond) jerks with a cortical discharge on the electroencephalogram (EEG). Historically, it is not clear if there is a neurobiological distinction between the 2 phenomena, and therefore we decided to include them both in the current myoclonus nomenclature.

The first 2 papers of the task force included the proposed nomenclature for genetic parkinsonism, dystonia, autosomal‐dominant and ‐recessive cerebellar ataxia, hereditary spastic paraplegia, paroxysmal movement disorders, neurodegeneration with brain iron accumulation, and primary familial brain calcification.^1^, ^2^ Here, we present the genetically determined myoclonus syndromes nomenclature based on the same principles, criteria, and recommendations.

## Methods

### Inclusion

Our recommendations are based on a systematic literature search. All articles regarding genetic causes of myoclonus syndromes were identified by a PubMed, Online Mendelian Inheritance in Man, and Textbook search, including all the additional relevant references cited in the articles found. The key search terms “myoclonus,” “myoclonic epilepsy,” and “startle” were used in combination with the term “genetic causes.” For the period to June 2015, we used our previously published systematic review with the same search terms.^8^ In addition, an identical search was performed for the period between June 2015 and October 2018 to identify newly discovered genes. All reviewed articles and abstracts were restricted to those published in English.

Following the recommendations of the task force, the criteria for gene inclusion are that mutations in the gene must be causative (ie, risk factor genes were excluded), and myoclonus must be a prominent feature. In determining the pathogenicity, no specific threshold for the level of penetrance of a mutation was designated by the Movement Disorder Society (MDS) Task Force and was determined for each gene based on standards prevailing in the field. In the field of myoclonus, we decided that genes related to myoclonus or myoclonic epilepsy with medium or low penetrance were excluded. In Table 1 we included genetic disorders DYT‐*ANO3* and CHOR‐*NKX2‐*1, although the penetrance of these genes is reduced. The reason to include them is that the previous nomenclature of the MDS Task Force decided to include lower penetrance, as it is more common in dystonic syndromes and these 2 genes present with the clinical syndrome of myoclonus‐dystonia.

**Table 1:** The proposed new list of genetically determined myoclonus syndromes

	New designation	Name	Myoclonus	Ataxia	Dystonia	Epilepsy	Cognitive problems	Clinical clues	Myoclonic subtype	OMIM	Inheritance pattern	Locus symbol
Prominent myoclonus syndromes												
	MYC‐*CLN3* 11	CLN3 disease	+	−/+	−	++	++	Juvenile onset, parkinsonian signs, retinal degeneration, neuropsychiatric symptoms	CM^a^	607042	AR	CLN3
	MYC‐*CLN5* 12	CLN5 disease	++	−/+	−	++	++	Late‐infantile onset, blindness	CM^a^	608102	AR	CLN5
	MYC‐*CLN6* 13	CLN6 disease	++	+/++	−	++	++	Early juvenile or adult^14^ onset, visual failure	CM^a^	606725	AR	CLN6
	MYC‐*CLN8* 15	CLN8 disease	++	+/++	−	++	++	Late infantile onset, retinopathy	CM^a^	607837	AR	CLN8
	MYC‐*DNAJC5* 16	CLN4 disease	++	+/++	−	++	++	Adult‐onset	CM^a^	611203	AD	CLN4
	MYC‐*GLRA1* 17; MYC‐*SLC6A5* 18; MYC‐*GLRB* 19	Hyperekplexia	+	−	−	−	−	Generalized stiffness at birth and following startle, neonatal tonic cyanotic attacks, periodic limb movement during sleep, and hypnagogic myoclonus	BSM	138491 604159 138492	AD, AR AD, AR AR	HKPX1 HKPX3 HKPX2
	MYC‐*KCNC1* 20	MEAK	++	++	−	+	−/+	−	CM	176258	AD	None
	MYC‐*PRICKLE1* 21	EPM 1B	++	++	−	+	−/+	Upward gaze palsy	UN	608500	AR	None
	MYC‐*SAMD12*,^c^ MYC‐*RAPGEF2* 22	FCMTE	+	−	−	+/++	−/+	Adult‐onset, anxiety, and depression^23^	CM	618073 609530	AD	None
	MYC‐*SCARB2* 24	AMRF syndrome	++	+/++	−	+/++	−/+	Tremor, renal failure, peripheral neuropathy	CM	602257	AR	None
	ATX/HSP‐*FOLR1* 25	Cerebral folate transport deficiency	−/+	++	−	++	++	Chorea, drop attacks^26^	UN	136430	AR	None
	*CARS2* 27	CARS2	−/+	−	−	++	++	Tetraparesis, visual and hearing impairment, areflexia, hypotonia^28^	UN	612800	AR	None
	*CHD2* 29	CHD2 encephalopathy	−	−	−	+/++	+/++	Photosensitivity, multiple seizure types of which atonic‐myoclonic‐absence is most common^30^	CM	602119	AD	None
	CUX2^31^	Myoclonic DEE	−	−	−	++	++	Infantile‐onset myoclonic and absence seizures, stereotypies and dyskinesias	CM	610648	AD	None
	*GLDC* 32; *AMT* 33	Classic non‐ketotic hyperglycinemia	−	−	−	++	++	Neonatal onset: progressive lethargy, hypotonia	CM	238300 238310	AR	None
	mt‐*MTTK* 34 d	MERRF	−	+	−	++	−/+	Muscle weakness, hearing loss, peripheral neuropathy, optic atrophy, axial lipomas, and variable other neurological manifestations (heterogeneous disease, multiple genes associated with phenotype)^35^	CM	590060	Mt	None
	*PIGA* 36	MCAHS2	−	−	−	++	++	Dysmorphic features, neonatal hypotonia	CM	311770	XLR	None
	*POLG* 37	POLG‐related disorders	−/+	−/+	−/+	++	++	Parkinsonism, chorea, migraine, stroke‐like episodes, hearing and visual impairment, myopathy, neuropathy, endocrine and gastrointestinal disorders	UN	174763	AD or AR	None
	*SCN1A* 38 e	Dravet syndrome	−/+	−/+	−	++	+/++	Febrile and prolonged seizures with alternating pattern	CM	607208	AD	None
	*SERPINI1* 39, 40	FENIB	−	−/+	−	++	++	−	CM	602445	AD	None
	*SLC6A1* 41	Doose syndrome	−	−	−	++	+	Atonic drop attacks	CM	137165	AD	None
	*TBC1D24* 42	TBC1D24‐related disorders	−/+	−/+	−/+	+/++	+/++	Variable types of seizures, muscle hypotonia, extrapyramidal signs, hearing and visual loss^43^	UN	613577	AR	None
Combined myoclonus syndromes^f^												
	MYC/ATX‐*CSTB* 44	Unverricht‐Lundborg	++	++	−	+	−/+	Periodicity of symptoms^45^	CM	601145	AR	None
	MYC/ATX‐*EPM2A* 46	Lafora disease	++	++	−	++	++	Focal visual seizures, drop attacks, psychosis^47^	CM	607566	AR	None
	MYC/ATX‐*GOSR2* 48	North Sea PME	++	++	−	+/++	−/+	Scoliosis, areflexia, pes cavus, syndactyly, drop attacks	CM	614018	AR	None
	MYC/ATX‐*KCTD7* 49	EPM 3	++	++	−	++	++	Pyramidal signs, micorcephaly^50^	UN	611726	AR	None
	MYC/ATX‐*NEU1* 51	Sialidosis	++	++	−	−/+	+/++	Cherry‐red spots^52^	CM	608272	AR	None
	MYC/ATX‐*NHLRC1* 53	Lafora disease	++	++	−	++	++	See MYC‐EPM2A	CM	608072	AR	None
	MYC/ATX‐*TPP1* 54	CLN2 disease	++	++	−	++	++	Late infantile onset, retinopathy, spasticity, hypotonia, extended vegetative state	CM^a^	204500	AR	CLN2
	MYC/DYT‐*SGCE* 55	Myoclonus‐dystonia (M‐D)	+	−	+	−	−	M‐D predominantly in upper body, psychiatric disorders	SCM	604149	AD	DYT11
	MYC/DYT‐*KCTD17* 56	Myoclonus‐dystonia	+	−	+	−	−	M‐D predominantly in upper body, laryngeal involvement can occur, psychiatric symptoms	SCM	616386	AD	None
Disorders that usually present with other phenotypes but can manifest as a prominent myoclonus syndrome												
												
	ATX‐*ATM* 57	Variant Ataxia‐telangiectasia	+	+	++	−	−/+	M‐D phenotype, chorea^58^ Systemic abnormalities: immunodeficiency, malignancies, and oculocutaneous telangiectasias	SCM	607585	AR	None
	ATX‐*ATN1* 59 g	DRPLA, PME phenotype	+/++	++	−	+/++	++	PME phenotype especially in patients with age of onset < 20 years. Other phenotypes are an ataxochoreoathetoid form and a pseudo‐Huntington form	CM	607462	AD	None
	ATX‐*NPC1* 60	Niemann‐Pick type C	++	++	−/+	−/+	+/++	PMA‐phenotype, chorea, and tremor,^61^ hepatosplenomegaly, vertical supranuclear gaze palsy	CM	607623	AR	None
	ATX‐*PRKCG* 62 g	SCA 14	+	+	−/+	−	−/+	M‐D phenotype, sensory loss, hyperactive tendon reflexes, depression^63^	SCM	176980	AD	SCA14
	DYT‐*ANO3* 64	Tremorous cervical dystonia	+	−	++	−	−	M‐D predominantly in upper body, tremor	SCM	610110	AD	DYT24
	CHOR/DYT‐*ADCY5* 65	FDFM	+	−	+	−	−/+	M‐D phenotype with episodic mixed hyperkinetic disorder of the face characterized by myoclonus‐chorea,^66^ axial hypotonia	UN	600293	AD	None
	CHOR‐*HTT* 67	Juvenile Huntington's disease	++	++	−	−/+	+/++	Behavioral symptoms and parkinsonian signs^68^	CM	613004	AD	None
	CHOR‐*NKX2‐1* 69	Benign hereditary chorea	++	+	+/++	−	+	M‐D phenotype, chorea more prominent at young age, in adult life myoclonus most disabling if present. Tics, brain‐lung‐thyroid syndrome.	UN	600635	AD	None
	HSP‐*KIF5A* 70	Neonatal myoclonus	++	−	−	−/+	++	Neonatal onset. Eye movement abnormalities, apnea, ptosis, optic nerve pallor, hypotonia. Leukoencephalopathy may be seen.^71^	UN	602821	AD	SPG10
	HSP‐*SACS* 20	ARSACS	++	++	−	++	++	Pyramidal signs^72^	CM^a^	604490	AR	None
	PARK‐*GBA* 73	Neuronopathic Gaucher disease	+/++	+/++	−	++	++	Spasticity, horizontal gaze abnormalities, visceral involvement^74^	CM^a^	606463	AR	None
	*APP* 75	Familial Alzheimer's disease	+	−/+	−	+	++	−	CM	104760	AD	None
	*ASAH1* 76	Spinal muscular atrophy	++	−	−	++	−/+	Progressive lower motor neuron disease manifestations	CM^a^^,^ h	613468	AR	None
	*CSNK2B* 77	*CSNK2B*‐related disorders	−	−	−	++	+	Infantile onset of myoclonic seizures. Speech and language disorder.	CM	115441	AD	None
	*CTSA* 78	Galactosialidosis	++	++	−	+/++	++	Coarse facies, vertebral changes, cherry‐red spots, corneal clouding, absence of visceromegaly, angiokeratoma^79^	CM	613111	AR	None
	*FARS2* 80	*FARS2‐*related disorders	−	−	−	++	++	Early infantile onset of myoclonic seizures, GTCS, and infantile spasms.	CM	611592	AR	None
	*PRNP* 81 i	Familial Creutzfeldt‐Jakob disease	++	++	−	−/+	++	Chorea, visual impairment, akinetic mutism, sleep disturbances, psychiatric disorders, peripheral neuropathy^82^	CM & SCM	176640	AD	None
	*PSEN1* 83	Familial Alzheimer's disease	+	−/+	−	+	++	Spastic paraparesis, rigidity, behavioral symptoms, language and dysexecutive deficits^84^	CM	104311	AD	None
	*RPS6KA3* 85	Coffin‐Lowry syndrome	+	−	−	−	+	Stimulus‐induced drop episodes,^86^ dysmorphism, progressive skeletal changes, hearing loss, mitral valve deformity	UN	300075	XLD	None
	*SLC2A1* 87	Glucose transport type 1 deficiency	−	−/+	−	++	+/++	Myoclonic, myoclonic‐astatic, GTC, and absence seizures starting in early up to middle childhood. Other phenotypes include paroxysmal exertion‐induced dyskinesia, absence epilepsy or episodic choreoathetosis, and spasticity.^88^	CM	138140	AD	None
	*SYNGAP1* 89	*SYNGAP1*‐associated intellectual disability and epilepsy	−	−/+	−	+/++	+/++	Early infantile onset of drop attacks, massive myoclonic jerks, and (myoclonic)‐absence seizures. Hypotonia, behavioral disorder, ASD, orthopedic problems.	CM	603384	AD	None
	*UBE3A* 90	Angelman syndrome	+	−/+	−	++	++	Myoclonic, myoclonic absence, and myoclonic‐tonic seizures in early childhood; nonepileptic myoclonus first presenting in adolescence. Sleep dysfunction, absent or limited expressive language.^91^	CM^a^	601623	b	None
	mUDPC7^92^	Silver‐Russell syndrome	+	−	+	−	−	Growth retardation, dysmorphism, M‐D predominantly located in upper body	UN	180860	IC	None

++, Severe/progressive presentation of symptom; +, mild presentation of symptom; −/+, symptom can be present or absent; ‐ symptom is absent.

AMRF, action myoclonus renal failure; ARSACS, autosomal‐recessive spastic ataxia of Charlevoix‐Saguenay; BSM, brain stem myoclonus; CM, cortical origin of myoclonus; DEE, developmental and epileptic encephalopathy; DRPLA, dentate‐rubro‐pallido‐luysianatrophy; EPM, progressive myoclonus epilepsy; FCMTE, familial cortical myoclonic tremor with epilepsy; FDFM, familial dyskinesia with facial myokymia; FENIB, familial encephalopathy with neuroserpin inclusion bodies; ICs, isolated cases; MCAHS2, multiple congenital anomalies‐hypotonia‐seizures syndrome‐2; M‐D, myoclonus‐dystonia; MEAK, myoclonus epilepsy and ataxia from potassium (K^+^) channel mutation; MERRF, myoclonic epilepsy with ragged red fibers; SCM, subcortical origin of myoclonus; UN, myoclonic subtype is unknown; XLD, X‐linked dominant; XLR, X‐linked recessive.

a Myoclonic subtype could not be assigned according to the official criteria stated by Zutt et al (2018)^83^; therefore, the subtype stated in the literature was adopted but accentuated as presumed using an asterisk.

b Loss of the maternally inherited *UBE3A* gene.

c Recently, authors have proven the pentanucleotide repeat TTTCA (and TTTTA) to be causative of FCMTE in the intron of MYC‐*SAMD12* and MYC‐*RAPGEF2*.^12^ Although the authors believe the intronic pentanucleotide repeat to be pathogenic irrespective of the gene, we have stated the 2 genes that have been confirmed in the literature.

d The following additional genetic mutations are able to cause MERRF: mt‐*MTTL11* (OMIM 590050), mt‐*MTTH1* (OMIM 590040), mt‐*MTTS11* (OMIM 590080), mt‐*MTTS21* (OMIM 590085), mt‐*MTTF1* (OMIM 590070), mt‐*MTTW* (OMIM 590095)1.

e The following genes have been reported to cause a DS‐like phenotype by at 2 independent research groups: *SCN1B* (OMIM 600235), *PCDH19* (OMIM 300460), *GABRA1* (OMIM 615744).

f The phenotype of a combined myoclonus syndrome is characterized by multiple predominant movement disorders including myoclonus.

g Because of recent suggestions of the Task Force Nomenclature, the previously proposed prefix SCA for autosomal‐dominant ataxias was replaced by ATX, resulting in the replacement of prefixes of 2 genes, *ATN1* and *PRCKG*. SCA‐*ATN1* has been changed to ATX‐*ATN1* and SCA‐*PRKCG* to ATX‐*PRKCG*.

h Patients diagnosed with a genetic defect of *ASAH1* were described by Topaloglu et al (2016) as having subcortical myoclonic epileptiform abnormalities. However, based on the clinical characteristics we suspect a cortical origin of the myoclonic jerks and have classified this gene accordingly.

i Opposed to the previously assigned prefix CHOR in CHOR‐*PRNP*, the prefix CHOR was removed, and the name was altered to *PRNP*, as this gene causes multiple phenotypes including myoclonus and in which chorea only dominates in a minority of cases.

Cognitive problems include both cognitive decline and psychomotor retardation. The myoclonic subtype was determined unknown if neither an official myoclonic subtype could be assigned or a myoclonic subtype was stated in the literature.

Prominent myoclonus was present if either (1) the literature stated that myoclonic jerks were a prominent feature of the phenotype, (2) the myoclonic jerks were the main reason for disability, and/or (3) the myoclonic jerks were the main focus of treatment. In addition to this, the predominance of myoclonus in the disorder had to be confirmed in the literature by a second independent group of researchers.^1^

This adjudication process included 2 persons (S.V. and R.Z.). All genes included in the new nomenclature were reviewed by 6 experts within the field of myoclonus to reach a broadly supported consensus (H.S., J.C., S.B., P.T., T.K., M.T.).

### Classification

Following the recommendation of the task force and to guide clinicians in daily practice, the genetic disorders were allocated based on clinical presentation into 1 of the following 3 groups: (1) *prominent myoclonus syndromes*, genetic disorders that present with prominent myoclonus in the majority of cases; (2) *combined myoclonus syndromes*, genetic disorders that present with prominent myoclonus and another prominent movement disorder (eg, dystonia/ataxia) in the majority of cases; and (3) *disorders that usually present with other phenotypes but can manifest as a prominent myoclonus syndrome*, genetic disorders that present with prominent myoclonus only in a minority of cases as part of the phenotypic spectrum of this disorder.

### Prefixes

In accordance with the recommendations of the task force, the prefix MYC was given to genes in which myoclonus is a prominent feature in the majority of the patients. In addition, we added a second prefix to genes and consequently allocated it to the subgroup *combined myoclonus syndromes*, in which another movement disorder is an additional prominent feature, resulting in a double prefix if both movement disorders are prominent (eg, MYC/ATX‐*GOSR2*). Overlapping genes with double prefixes were discussed among the appropriate experts from the MDS Task Force to reach consensus. The symbol prefix is followed by the gene name. For clarity and to allow comparison with former classifications, we provided the old locus symbol (eg, DYT11) in the last column of Table 1, when appropriate. Genes that present with myoclonic epilepsy were not given any prefix, because the dominant feature of the phenotype is epilepsy rather than a movement disorder.

### Additional Clinical and Electrophysiological Items

A brief description of the clinical presentation of disorders linked to each gene is listed in Table 1 with special emphasis on the most common accompanying signs and symptoms including ataxia, dystonia, cognitive problems, or epilepsy. Furthermore, we added the myoclonic anatomical subtype, cortical or subcortical (ie, noncortical), if known, for each genetic disorder based on reported clinical and electrophysiological features to further improve the classification of myoclonus. Experts have argued against the term “subcortical” myoclonus, as its anatomical origin is still undetermined; however, the term “subcortical” myoclonus will still be used in the new nomenclature because of the absence of a widely supported alternative.^7^ See Supplementary Table 1 for the anatomical classification criteria for myoclonus.^93^

## Results

### Gene Selection

**Figure 1:**
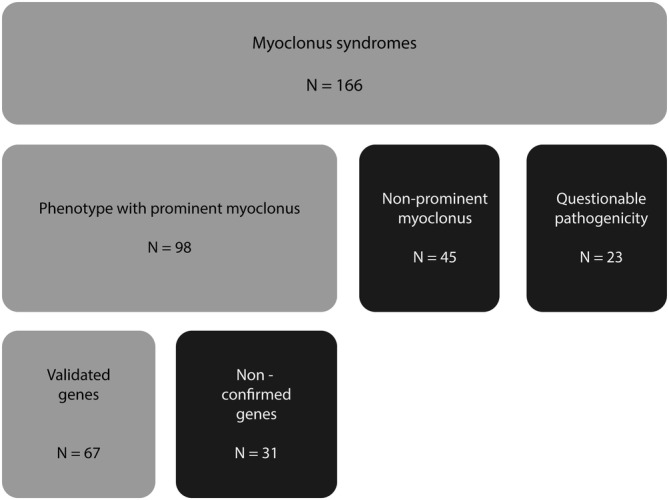
In and exclusion of genes associated with myoclonus syndromes.

One hundred sixty‐six genes linked to a myoclonus syndrome were found in the systematic literature review. An extensive overview of all genes associated with myoclonus with reason for inclusion or exclusion can be found in Supplementary Table 3, and see Figure 1 for an overview. Nighty‐nine genes were excluded because of the absence of prominent myoclonus (n = 45), lack of confirmation of the phenotype with prominent myoclonus by a second independent research group (n = 31), and questionable pathogenicity (n = 23).

Sixty‐seven genes were included in the new nomenclature for myoclonus syndromes (see Table 1). (1) In the subgroup *prominent myoclonus syndromes*, 35 genes were included; (2) in the subgroup *combined myoclonus syndromes*, 9 genes were included; and (3) in the subgroup *disorders that usually present with other phenotypes but can manifest as a prominent myoclonus syndrome*, 23 genes were included.

### Prefix Allocation

The locus symbol prefix MYC was assigned to 22 genes. Genes in which the predominant phenotype showed wide heterogeneity or was dominated by epileptic or nonmotor symptoms were not assigned any prefix. For myoclonus epilepsy with ragged red fibers syndrome, only the most frequent causative gene (mt‐*MTTK*) is listed. The remaining causative genes are stated in the caption of Table 1, as they are associated with a similar phenotype as mt‐*MTTK*.

### Additional Clinical and Electrophysiological Clues

The following most common accompanying signs and symptoms observed overall were cognitive decline in 90% (n = 60), epilepsy in 82% (n = 55), ataxia in 61% (n = 41), ataxia and dystonia in 9% (n = 6), and dystonia in 7% (n = 5). The anatomical location of myoclonic origin could be allocated in 64% of genes (n = 43) because of support of strong electrophysiological data, and in the cortex in 51% (n = 34), noncortical areas in 12% (n = 8), and both cortical and noncortical areas in 1% (n = 1) of all genes. Three of the 8 genes with jerks originating from noncortical areas were classified as originating from the brain stem (hyperekplexia).

## Discussion

In this article we propose a nomenclature of genetically determined myoclonus according to the new naming system presented by the MDS Task Force.^1^ This myoclonus list currently includes 67 genes. Thirty‐five genes presented with prominent myoclonus syndromes, 9 with combined myoclonus syndromes, and 23 with disorders that usually present with other phenotypes but can manifest as a prominent myoclonus syndrome. Co‐occurrence of movement disorders, especially ataxia and dystonia, was seen in almost all except for familial cortical myoclonus tremor with epilepsy (FCMTE, or BAFME, benign adult familial myoclonus epilepsy), hyperekplexia, and (myoclonic) epileptic encephalopathies. Epilepsy and cognitive decline were the most frequently observed accompanying clinical features for the disorders listed in this new nomenclature.

The literature search detected 166 genes linked to a myoclonus syndrome, but only 67 were used for the nomenclature list. Filtering using strict criteria (independent confirmation and predominant myoclonus) to arrive at a list of confirmed entities that can present with predominant myoclonus is meant to help the clinician with the selection of test procedures and assist in the interpretation of results of genetic testing.^2^ In our opinion, the requirement for independent confirmation by a second research group is an important criterion, as it diminishes erroneous genotype‐phenotype linkages. At present, with the widespread use of NGS in research and clinical diagnostics, many potentially new myoclonus genes are reported. Still, we had to exclude 31 genes (19%) that require validation. A significant proportion of patients with myoclonus syndromes still remain unsolved (progressive myoclonus ataxias in 36%^94^ and progressive myoclonus epilepsies in 28%^95^), in which excluded genes could be considered.

A new clinical diagnostic approach in patients with myoclonus has recently been described.^8^ After establishing that the myoclonus in a patient has a genetic cause, Table 1 can be used as a diagnostic framework for physicians in clinical practice to select candidate genes for individual patients based on the absence or presence of accompanying signs and symptoms.

FCMTE/BAFME is the only genetically determined myoclonus syndrome with relatively pure myoclonus, although it is accompanied by infrequent epilepsy in a majority of but not all patients. This genetic disorder is caused by 2 recently confirmed genes (MYC‐*SAMD12* and MYC‐*RAPGEF2*) with intronic expansions of noncoding TTTCA and TTTTA pentanucleotide repeats. It presents with a phenotype of benign CM with infrequent tonic‐clonic and sometimes focal seizures. RNA‐mediated toxicity resulting in diffuse loss of Purkinje cells in the cerebellum is suggested to be the underlying pathogenesis of this disorder.^96^, ^97^ The potential role of the cerebellum in CM has been pointed out multiple times in the literature, supported by the frequent phenotypical co‐occurrence of CM and cerebellar ataxia.^98^

*Ataxia* is the most common accompanying movement disorder in myoclonus syndromes (24 genes). Almost all patients in whom the genetic disorder consists of a combination of ataxia and myoclonus present with the clinical syndrome of progressive myoclonus ataxia (PMA) or progressive myoclonus epilepsy (PME). The most common and best characterized are Unverricht‐Lundborg disease (MYC/ATX‐*CSTB*), Lafora disease (MYC/ATX‐*EMP2A*), neuronal ceroid lipofuscinosis (multiple genes), sialidosis (MYC/ATX‐*NEU* 1), and dentatorubral pallidoluysian atrophy (ATX‐*ATN1*).^99^

The anatomical origin of myoclonus in most patients with ataxia is thought to be cortical. Clinically, cortical myoclonic jerks present typically in the distal limbs and face, jerks are provoked by action and are stimulus sensitive.^93^ Of the genetic disorders in which ataxia and myoclonus co‐occur, we found that cortical origin was supported by strong electrophysiological evidence in 54% (n = 14), and it was suspected in 33% (n = 8). Mechanistic hypotheses for cortical myoclonus include: (1) loss of Purkinje cells with astrocytosis, resulting in disinhibition via the cerebello‐thalamico‐cortical pathway, (2) neuronal cell loss in the dentate nuclei leading to impaired cerebellar projections to the cortex, or (3) a reduction in the concentration of γ‐aminobutyric acid (GABA)‐ergic synapses in the sensory‐motor cortex.^100^ On a molecular level, most genetic disorders presenting with both ataxia and myoclonus have impaired posttranslational modification of proteins to which certain neuronal groups might be particularly vulnerable compared with others.^100^ This could play a role in the characteristic phenotype of PMA, including a fixed order of signs, starting with ataxia, subsequently CM, and eventually by infrequent epilepsy.^94^

*Dystonia* is the second type of prominent movement disorder accompanying myoclonus. The combination of myoclonus and dystonia is known as myoclonus‐dystonia syndrome (M‐D). The classical myoclonus‐dystonia phenotype is based on genetic defects in the MYC/DYT‐*SGCE* gene in about 50% of cases.^101^ Other disorders that can give rise to a myoclonus‐dystonia phenotype include MYC/DYT‐*KCTD17*, DYT‐*ANO3*, ATX‐*PRKCG*, ATX‐*ATM*, CHOR/DYT‐*ADCY5*, CHOR‐*NKX2‐1*, and maternal uniparental disomy with regions of heterodisomy and isodysomy on chromosome 7 (mUPD7), which is based on the loss of function of the *SGCE* gene.

The anatomical locus of myoclonus in M‐D is subcortical. Clinically, the myoclonus and dystonia in M‐D are located mainly in the trunk and proximal upper limbs, and the myoclonus is not stimulus sensitive. The noncortical origin of the myoclonus is supported electrophysiologically in 5 genetic disorders presenting with M‐D (MYC/DYT‐*SGCE*, MYC/DYT‐*KCTD17*, DYT‐ANO3, ATX‐*ATM*, ATX‐*PRKCG*) and unknown in 2 others (CHOR/DYT‐*ADCY5* and CHOR‐*NKX2‐1*). The pathophysiology of subcortical myoclonus includes circuit abnormalities in the basal ganglia and involvement of the cerebellum. Disruptions in neurotransmission pathways have been hypothesized to play a role, particularly the unbalanced homeostasis of GABA, serotonin, and dopamine‐related pathways.^102^ In contrast to myoclonus of cortical origin, cortical excitability and intracortical inhibition were found to be normal or less profoundly disturbed.^103^

The overlap between types of accompanying movement disorders and the anatomical origins of the myoclonic jerks is remarkable. Currently, the anatomical origin can be assigned in only 64% of genetic disorders. We encourage movement disorder specialists to classify the subtype of myoclonus by a thorough clinical description (eg, distribution, stimulus sensitivity) of the myoclonic jerks and if possible electrophysiological testing (eg, corticomuscular coherence or jerk‐locked back‐averaging). We realize that availability of the tests varies considerably between centers and countries.^6^ However, the myoclonic subtype guides the clinician toward a more precise differential diagnosis (see Table 1) and effective treatment strategy,^104^ and it helps to unravel the pathogenesis of myoclonus by creating homogenous groups.

*Epilepsy* is an additional feature in 82% of myoclonus syndromes, presenting either as CM in combination with epilepsy or myoclonic jerks as part of a myoclonic seizure. It is only described in genes with jerks originating from the cortex, as mutations in genes linked to noncortical myoclonus (hyperekplexia, all M‐D syndromes, and Coffin‐Lowry syndrome) rarely present with epileptic manifestations. The distinction between myoclonus and (myoclonic) epilepsy can be difficult to make, and seemingly minor differences in terminology can create confusion. *Myoclonus* epilepsy is a condition in which CM, often continuously present, and epilepsy occur independently, whereas *myoclonic* epilepsy is an attack of generalized convulsions starting with myoclonic jerks or predominantly characterized by myoclonic jerks. Jerks in both CM and myoclonic epilepsy are associated with EEG polyspikes or spike/polyspike‐wave complexes before the onset of an EMG burst.^105^ Confusion is not only the case in clinical practice but also in the literature, making it difficult to interpret many of the clinical presentations described. For instance, the phenotype associated with MYC/ATX‐*GOSR2* has been called an epileptic syndrome with myoclonic seizures (progressive myoclonus epilepsy type 6) in articles from the field of epilepsy,^106^ as opposed to a syndrome with prominent cortical myoclonus in combination with epilepsy (progressive myoclonus ataxia) in articles from the field of movement disorders.^107^ Particularly in the fields of movement disorders and epilepsy, the phenotype is a decisive factor for further diagnostics, and inaccuracy of descriptions can lead to erroneous genotype‐phenotype relationships. Ongoing discussion and consensus meetings between experts in both fields are necessary to accomplish a consistent terminology with clear definitions that could easily be implemented in clinical practice.

*Cognitive problems* including cognitive decline and psychomotor retardation have been reported in all but 5 genetic disorders, MYC/DYT‐*SGCE*, MYC/DYT‐*KCTD17*, mUDP7 (based on loss of *SGCE*‐gene), DYT‐*ANO3*, and the hyperekplexias. Other nonmotor features, particularly psychiatric disorders and behavioral problems, are also being recognized as part of the phenotype of certain movement disorders (eg, dystonia). In disorders with cortical myoclonus, almost half the patients experience symptoms of depression or anxiety.^108^ Underestimation of these nonmotor features is likely, as we have only recently started considering this to be part of the phenotype. Future case descriptions of myoclonus syndromes should include details on cognition, psychiatric symptoms, and behavioral changes. The clinician should be aware of the high occurrence of nonmotor features in patients with myoclonus syndromes. These are features that impact the patient's life and his or her family, and they require proper guidance and counseling.^109^

Just as the presence of accompanying signs and symptoms can guide clinicians to a refined differential diagnosis, absence of an accompanying movement disorder proves a useful observation, as it points toward the related disorders, *hyperekplexia and myoclonic epileptic encephalopathies*. Hyperekplexia is characterized by 3 clinical symptoms: generalized stiffness at birth, excessive startle reflexes, and generalized stiffness following a startle. Genetic studies have shown mutations in different parts of the inhibitory glycine receptor complex, located in the postsynaptic membrane of glycinergic and mixed GABAergic neurons. Synaptic inhibition in the brain stem and spinal cord is impaired as a result of a defect in 1 of these 3 genes.^10^ With regard to the genes identified in epileptic encephalopathies with prominent myoclonic jerks, a majority of these disorders share a phenotype that includes early disease onset (in the first 18 months of life) and a progressive course resulting in refractory epilepsy and severe cognitive decline. However, some genetic disorders are extremely rare (eg, *CARS2*), and those phenotypes are likely to be expanded in the coming years.

### Conclusion

In collaboration with the MDS Task Force, we present a new nomenclature that includes 67 genetically determined myoclonus syndromes. As is apparent from this current list, numerous genes are linked to myoclonus syndromes, and prioritizing putative causative genes based on corresponding accompanying signs or symptoms and clinical clues could accelerate the identification of a molecular diagnosis in individual cases. Furthermore, it shows the additional value of electrophysiological testing in patients with myoclonus syndromes, as it may lead to a more refined differential diagnosis and therapeutic strategy. The current nomenclature can be used as a framework to add newly discovered genes in a systematic way and can be used for movement disorder (myoclonus) next‐generation sequencing diagnostics. In the near future, genetically determined myoclonus syndromes can be uploaded in the searchable online database, the Movement Disorder Society Genetic Mutation Database, MDSGene (http://www.mdsgene.org), to provide an online, browsable database of hereditary myoclonus syndromes.^110^

## Author Roles

1) Research project: A. Conception, B. Organization, C. Execution; 2) Statistical Analysis: A. Design, B. Execution, C. Review and Critique; 3) Manuscript: A. Writing of the first draft, B. Review and Critique.

S.V.: 1B, 1C, 3A, 3B.

R.Z.: 1A, 1B, 1C, 3B.

C.K.: 1A, 3B.C.M.: 1A, 3B.

S.F.B.: 3B.

J.N.C.: 3B.

H.S.: 3B.

T.J.K.: 1A, 1B, 1C, 3B.

M.A.J.T.: 1A, 1B, 1C, 3B.

## Full financial disclosure for the previous 12 months

C.K. is a medical adviser to Centogene, received honoraria from the Wellcome Trust Review Board and the Scientific Advisory Board of the Else Kroener Fresenius Foundation, and received grants from the Hermann and Lilly Schilling Foundation, the German Research Foundation, the BMBF, the German Research Foundation, the European Community, and intramural funds from the University of Luebeck. She receives royalties from the Oxford University Press and is employed at the University of Luebeck. C.M. is consultant for Acorda, received honoraria for teaching from EMD Serono and has received grants from the Michael J. Fox Foundation, Canadian Institutes of Health Research, and National Parkinson Foundation. She was site principal investigator for a clinical trial sponsored by the National Institutes of Health, International Parkinson and Movement Disorder Society and Parkinson Disease Foundation. She has received contracts from Horizon Pharma and is employed with the University Health Network. For this article, she received support from the International Parkinson and Movement Disorder Society. T.J.K. received grants from Metabolic Power Foundation, Metakids Foundation, and Ride4Kids Foundation (all nonprofit) for studying movement disorders in metabolic diseases. He received research grants from Actelion Pharmaceuticals (profit) for studying movement disorders in Niemann‐Pick‐C disease and received an honorarium for presenting at a sponsored meeting on Niemann‐Pick‐C. M.A.J.T. has received research grants from the Dystonia Medical Research Foundation, Stichting Wetenschapsfonds Dystonie Vereniging, Prinses Beatrix Foundation, Fonds NutsOhra, Jacques and Gloria Gossweiler Foundation, Fonds Psychische Gezondheid, and Phelps Stichting and unrestricted grants for education and for the national DystonieNet from Ipsen & Allergan Farmaceutics, Merz, Medtronic, and Actelion. S.V., R.Z., S.F.B., J.N.C., and H.S. have nothing to disclose.

## Supporting information


**Supplementary table 1** The electrophysiological and clinical features of myoclonus, its subtypes and mimic.
**Supplementary table 2**. The clinical and electrophysiological features of myoclonic jerks stated for each gene.

**Supplementary table 3** Overview of genes presenting with myoclonus
