# Supplementary material for: Nomenclature of Genetically Determined Myoclonus Syndromes: Recommendations of the International Parkinson and Movement Disorder Society Task Force
Source: Mov Disord. 2019 Oct 4;34(11):1602–13. doi: 10.1002/mds.27828 (PMC6899848; doi:10.1002/mds.27828)
Supplement: Supplementary file 1 — Supplementary table 1 The electrophysiological and clinical features of myoclonus, its subtypes and mimic. Supplementary table 2. The clinical and electrophysiological features of myoclonic jerks stated for each gene. [file MDS-34-1602-s001.docx]

**Supplementary table 1.** The electrophysiological and clinical features of myoclonus, its subtypes and mimic.

| Myoclonus and its subtypes | | Electrophysiological and clinical features^1^ |
| --- | --- | --- |
| Myoclonus |  | Abrupt muscle contraction or interruption of tonic muscle activity **(required)** |
|  |  | Synchronous contraction of agonist and antagonist muscles |
|  |  |  |
| Cortical |  | Burst duration of positive myoclonus <100ms |
|  |  | Multifocal/focal distribution |
|  |  | Affecting the face and distal limbs |
|  |  | Spontaneous, action-induced or stimulus-sensitive jerks |
|  |  | Presence of negative myoclonus |
|  |  | Positive cortical spike by back-averaging **(strong evidence)** |
|  |  | Positive cortico-muscular coherence **(strong evidence**) |
|  |  | Presence of giant somatosensory evoked potential  Presence of long loop transcortical reflex |
|  |  |  |
| Subcortical | Brainstem | Burst duration >100ms |
|  |  | Simultaneous rostral and caudal muscle activation at brainstem level |
|  | Myoclonus-Dystonia | Burst duration >100ms |
|  |  | Do not meet criteria of other categories |
|  | Opsoclonus-Myoclonus^2^ | Involuntary, arrhythmic, chaotic, multidirectional, fast eye movements |
|  |  | Multifocal myoclonus involving axial muscles and limbs |
|  | Reticular reflex myoclonus^3^ | Increased motor reflex with spreading of activity from the medulla oblongata both in rostral and caudal direction |
|  |  |  |
|  |  |  |
| Spinal | Segmental | Burst duration >100ms |
|  |  | Distribution according to one or two contiguous spinal segments **(required)** |
|  |  | My be periodic (1-2/min-240/min) |
|  | Propriospinal | Burst duration >100ms |
|  |  | Initiation in the mid thoracic segments followed by rostral and caudal activation **(required)** |
|  |  | Propagation with slow velocity (5-15 m/s) in cord **(required)** |
|  |  |  |
| Peripheral |  | Burst duration <50ms **(required)** |
|  |  | Large MUAPs **(required)** |
|  |  | Minipolymyoclonus or fasciculations/myokymia **(required)** |
|  |  | Accompanied by weakness/atrophy |
|  |  |  |
| Hyperekplexia^4,5^ | | Enhance startle reflex |
|  |  | Jerks followed by short-lasting generalized stiffness |
|  |  | Relatively delayed electromyographic response in the intrinsic hand and foot muscles |

**References**

1. Zutt R, Elting JW, van der Hoeven JH, Lange F and Tijssen MAJ. Myoclonus subtypes in tertiary referral center. Cortical myoclonus and functional jerks are common. Clin. Neurophysiol. 2017; **128**: 253–259.

2. Zutt R, van Egmond ME, Elting JW, et al. A novel diagnostic approach to patients with myoclonus. Nat. Rev. Neurol. 2015; **11**: 687–697.

3. Dreissen YEM and Tijssen MAJ. The startle syndromes: Physiology and treatment. Epilepsia 2012; **53**: 3–11.

4. Zutt R, Tijssen MAJ and Elting JW. Myoclonus. In: Parkinson Disease and other Movement Disorders. Edited by E Wolters. 1st ed. VU University Press 2014; pp 513–533.

5. Brown P, Rothwell JC, Thompson PD, Britton TC, Day BL and Marsden CD. The hyperekplexias and their relationship to the normal startle reflex. Brain 1991; **114 ( Pt 4)**: 1903–28.

**Supplementary table 2**. The clinical and electrophysiological features of myoclonic jerks stated for each gene.

| **Gene** | **(1)**  **Clinical characteristics of myoclonus** | **(1)**  **Clinical characteristics of myoclonic epilepsy** | **(2)**  **Electrophysiological characteristics of myoclonus or myoclonic epilepsy** | **(3)**  **Myoclonic subtype described in papers** | **(4)**  **Official myoclonic subtype in accordance with aforementioned criteria** | **(5)**  **Presumed myoclonic subtype if (4) not applicable** |
| --- | --- | --- | --- | --- | --- | --- |
| ADCY5 | Perioral twitches. And multifocal non-stimulus sensitive myoclonic jerks at rest and on posture, more prominent in the upper body. | N.A. | Bursts less than 100ms duration. Other article (Carecchio et al. 2017) burst 100-120ms in upper limbs and neck, without EEG correlate. | Origin of myoclonus is to be determined | Unknown | Unknown |
| ANO3 | Upper limb myoclonus | N.A. | Duration of myoclonus was about 250ms, with a variable frequency of 3 to 4 Hz at rest | Subcortical myoclonus (Stamelou et al. 2014) | Subcortical myoclonus | Subcortical myoclonus |
| APP & PSEN1 | Generalized myoclonus | N.A. | Jerk-locked back-averaging can detect a contralateral negative EEG potential preceding the jerks | Cortical myoclonus (Beagle et al. 2017) | Cortical myoclonus | Cortical myoclonus |
| ASAH1 | Proximal limbs, face, eyelid myoclonia. Action myoclonus. | Progressive myoclonic and atonic seizures with head drop or postural lapses in the upper arms. Brief myoclonic seizures without loss of consciousness | EEG showed multifocal epileptic foci, especially in central and occipital regions, secondary bilateral hypersynchrony besides generalized spikes and polyspike waves. EEG shows subcortical myoclonic epileptiform abnormalities sensitive to hyperventilation. | Myoclonic epilepsy and subcortical myoclonic epileptiform abnormalities | Unknown | Myoclonic epilepsy & unknown |
| ATM | Upper limbs, proximal or distal. Asymmetrical movements. Few had axial involvement. Non stimulus-sensitive. | N.A. | A wide range of discharge duration, the shortest being 20 ms and the longest being 385ms. | Subcortical myoclonus (Teive et al. 2018) | Subcortical myoclonus | Subcortical myoclonus |
| ATN1 | Myoclonus in upper extremities | Daily myoclonic epilepsy, stimulus-sensitive myoclonus. | Bilateral diffuse polyspike-and-wave complexes. Photic stimulation showed a photoparoxysmal response and provoked seizures. Positive back-averaging of EEG | Myoclonic epilepsy & cortical myoclonus | Cortical myoclonus | Cortical myoclonus & myoclonic epilepsy |
| CARS2 | Myoclonus of distal extremities and mouth. | Myoclonic epilepsy . Severe frequent myoclonic seizures | He developed multi- focal epileptiform discharges on EEG most prominent over the posterior quadrant, with slowing posteriorly and, at present, continuous multifocal high-amplitude epileptic discharges on EEG. | Myoclonic epilepsy. Myoclonus not further specified | Unknown | Myoclonic epilepsy & unknown |
| CHD2 | N.A. | Myoclonic-atonic seizures, absence, myoclonic jerks as seizure types. Myoclonus was both spontaneous and triggered by environmental photic stimuli (Thomas et al. 2015) Drop attacks | Interictal EEGs revealed slow back- ground activity associated with 2- to 4-Hz generalized epileptiform abnormalities enhancing during sleep. (Trivisano et al. 2015) | Myoclonic epilepsy | N.A. | Myoclonic epilepsy |
| CSNK2B | Multiple daily episodes of myoclonus in response to sonic stimulation (NOTE: interpreted as myoclonic epilepsy) | Epileptic myoclonus. Daily seizures next to myoclonus | EEG showed myoclonus of cortical origin with organized background activity | Cortical myoclonus; myoclonic epilepsy | Unknown | Myoclonic epilepsy |
| CSTB | Diffuse myoclonic jerks that predominate, movement-related, increases with stress. Reflex myoclonus, triggered by sensory stimulation, action myoclonus. | N.A. | Giant SEP, positive cortical-muscular coherence analysis. Brief EMG bursts (no value stated) | Cortical myoclonus (Franceschetti et al. 2016) | Cortical myoclonus | Cortical myoclonus |
| CUX2 | N.A. | Myoclonic and absence seizures. Seizures were frequent at onset with multiple seizures a day. | EEG was abnormal with most frequently showing generalized spike-wave or polyspike-wave. Focal features included temporal discharges, occipital discharges, multifocal discharges, hypsarrhythmia and focal slowing. Background slowing. Myoclonic seizures were recorded with GSW or GPSW. (Chatron et al. 2018) | Myoclonic epilepsy | N.A. | Myoclonic epilepsy |
| EPM2A & NHLRC1 | Action and stimulus-sensitive myoclonus. Myoclonic jerks next to generalized seizures. Positive and negative myoclonus. | Myoclonic absence seizures | Electrophysiological investigations can reveal aberrant integration of somatosensory stimuli and cortical hyperexcitability. Giant SEP is present. Significant coherent EEG-EMG activity during action myoclonus and positive JLBA. | Cortical myoclonus (Zutt et al. 2018) | Cortical myoclonus | Cortical myoclonus |
| FARS2 | N.A. | Hemiclonic, GTC and myoclonic seizures. Myoclonic seizures of the trunk or both arms and legs. | Multifocal epileptiform discharges | Myoclonic epilepsy | N.A. | Myoclonic epilepsy |
| FOLR1 | Asynchronous myoclonic jerks. Multifocal jerks. | Myoclonic tonic attacks with drop attacks provoked by touch. | The multifocal myoclonic jerks on electromyographic recording (deltoids) were not associated with EEG activity. (Toelle et al. 2013) | None stated | Unknown | Unknown & myoclonic epilepsy |
| GBA | Synchronous involuntary jerks stated as myoclonus. Continuous myoclonus. | Myoclonic seizures | Multifocal seizures, epileptiform activity, giant SEP | Myoclonic epilepsy, cortical myoclonus (Park et al. 2003) | Unknown | Myoclonic epilepsy & cortical myoclonus |
| GLDC & ATM | N.A. | Myoclonic bursts. Sudden, generalized contractions of axial, craneo-cervical and proximal-limb musculatures, which appeared spontaneously or after diverse stimuli, occasionally as an isolated episode, but more frequently as prolonged clusters (Pardal-Férnandez et al. 2009) | Suppression-burst pattern. At times, the paroxysmal bursts were roughly synchronous with myoclonus, whereas at other times, fragmented myoclonus appeared without EEG associations. (Suzuki et al. 2010)  EEG changed from a burst-attenuation pattern, with interictal bursts of medium amplitude, to a burst-suppression pattern five days later. (Pardal-Férnandez et al. 2009) | Myoclonic epilepsy | N.A. | Myoclonic epilepsy |
| GLRA1 & SLC6A5 & GLRB | Excessive startle reflex following unexpected stimuli, particularly auditory. | N.A. | Increased motor reflex with spreading of activity both up the brainstem and down the spinal cord | Brainstem myoclonus | Brainstem myoclonus | Brainstem myoclonus |
| GOSR2 | Action myoclonus, photosensitive generalized myoclonus, worsened with action or emotional stress | Drop attacks | Giant SEP, spikes or spike-wave forms preceding myoclonic jerks, supporting cortical reflex myoclonus. Time-locked association between the myoclonic jerks and EEG.  Burst duration below 100ms (Veen et al. 2018) | Cortical reflex myoclonus.  Cortical myoclonus. | Cortical myoclonus | Cortical myoclonus |
| HTT | Action and rest myoclonus, with a quasi-rhythmic course | N.A. | Brief EMG bursts (<50ms), JBLA revealed the presence of an EEG correlate. EEG-EMG coherence revealed significant peaks in the beta band. No giant SEP | Cortical myoclonus | Cortical myoclonus | Cortical myoclonus |
| KCNC1 | Action-induced myoclonus | N.A. | On EEG generalized polyspike, polyspike-wave and sometimes spike-wave discharges, photosensitivity. Jerks without obvious EEG correlates and showing a quasirhythmic time course. EEG-EMG revelaed a clear coherence peak in the beta band. Giant SEP and enhanced C-reflexes present. | Cortical-reflex myoclonus | Cortical myoclonus | Unknown |
| KCTD7 | Continuous multifocal myoclonus, aggravated by action and posture. Negative focal myoclonus | Myoclonic seizures | Multifocal and/or generalized spike-waves associated with an excess of slow activity. Intermittend light stimulation evoked generalized or posterior epileptiform discharges. Action myoclonus was not associated with concomitant epileptiform discharges. | Myoclonic epilepsy | Unknown | Myoclonic epilepsy & unknown |
| KCTD17 | Myoclonus predominantly in the arms. Low amplitude brief myoclonus. | N.A. | Back averaging was performed; myoclonus was of subcortical origin lasting 100–110 ms. | Subcortical myoclonus | Subcortical myoclonus | Subcortical myoclonus |
| KIF5A | Nearly continuous non-rhythmic, large amplitude jerks occurring singly or in brief clusters of 2-3 jerks. (Duis et al. 2016)  Stimulus-sensitive myoclonic jerks (Rydzanicz et al. 2017) | N.A. | No epileptiform abnormalities on EEG. | May be of spinal cord origin due to lack of EEG abnormalities (Duis et al. 2016) | Unknown | Unknown |
| MTTK & MTTL1 & MTTH & MTTS1 & MTTS2 & MTTF & MTTW | N.A. | Myoclonic epilepsy | Widespread paroxysmal abnormalities visible on EEG. | Suspected cortical myoclonus (Ganos et al. 2014)  Myoclonic epilepsy | N.A. | Myoclonic epilepsy |
| mUDPC7 | Action-induced myoclonus of upper limbs. Multifocal myoclonus. | N.A. | generalized spike-wave discharges but no electrographic change during the periods of myoclonus | None stated | Unknown | Unknown |
| NEU1 & CTSA | Multifocal myoclonus, high-frequency | N.A. | Subtle but highly rhythmic myoclonus, EEG consists of a discharge of fast activity, high cortico-muscular coherence, giant SEP, enhanced long-loop reflexes (C-reflex) | Cortical myoclonus | Cortical myoclonus | Cortical myoclonus |
| CLN3 & CLN5 & CLN6 & CLN8 & CLN4 & CLN 2 | Brief duration, multifocal, action-induced | N.A. | Presence of giant somatosensory evoked potential | Cortical myoclonus (Ganos et al. 2014 & Nita et al. 2016) | Unknown | Cortical myoclonus |
| NKX2-1 | Proximal myoclonus, as it is present in the trunk (Balicza et al. 2018 & Koht et al. 2016)  Neck, upper limbs and trunk (Asmus et al. 2007) | N.A. | EMG proven myoclonus, not further specified (Gras et al. 2012) | None stated | Unknown | Unknown |
| NPC1 | Myoclonus in trunk and extremities, both negative and positive myoclonus. Distal distribution. | N.A. | Abundant erratic cortical myoclonus . Intermittent light stimulation were related to generalized polyspikes. Short EMG burst duration <100ms. Positive coherence analysis and back-averaging | Cortical myoclonus | Cortical myoclonus | Cortical myoclonus |
| PIGA | Myoclonus NOTE: interpreted as myoclonic epilepsy | Severe myoclonic seizures | Myoclonic seizures with suppression bursts on EEG (Kato et al. 2014) | Myoclonic epilepsy | N.A. | Myoclonic epilepsy |
| POLG | Myoclonus, in some palatal myoclonus. | Generalized myoclonic seizures, involving limbs, shoulders, or necks. | Electro-encephalographic seizures | Myoclonic epilepsy | Unknown | Myoclonic epilepsy & unknown |
| PRICKLE1 | Action myoclonus affecting the limbs and bulbar muscles and spontaneous myoclonic jerks sometimes visible in facial muscles and in the limbs at rest. | Myoclonic seizures, tonic-clonic seizures. TCS often nocturnal. | EEG showed generalized spike-wave or poly-spike wave discharges and photosensitivity. | Myoclonic epilepsy | Unknown | Myoclonic epilepsy & unknown |
| PRKCG | Upper limb myoclonus, multifocal myoclonus. Located both proximal and distal. Increasing with action. Both positive and negative can be present | N.A. | EEG monitoring revealed no focal disturbances or epileptic phenomena, and no cortical correlates of the jerky arm movements were noted. Somatosensory evoked potentials (SSEPs) were normal. | Subcortical origin (Visser et al. 2007) | Subcortical myoclonus | Subcortical myoclonus |
| PRNP | Periodic myoclonus occurring every 0.5-2sec, involving upper limbs. At rest and during active movements. | N.A. | Positive EMG burst lasting 54.1 ± 15.8 milliseconds. Jerks always time-locked with PSWCs. JLBA (44–212 epochs) revealed that the onset of the PSWC waveform consistently preceded that of the EMG‐bursts recorded on the wrist extensor by 15‐68 milliseconds. Negative myoclonus. Some patients show dystonic myoclonus >200ms, appropriate detection of individual burst-onset and the application of JLBA not possible. Giant SSEP present. | Cortical myoclonus and subcortical myoclonus (Binelli et al. 2010) | Cortical myoclonus & subcortical myoclonus | Cortical myoclonus & subcortical myoclonus |
| RAPGEF2 & SAMD12 | Myoclonic tremor (cortical tremor); posture and rest myoclonus in hand (also legs, face, trunk), stimulus-sensitive. | Myoclonic-atonic, tonic-clonic and mostly myoclonic-tonic-clonic; | Focal spikes of multiple origin includ- ing occipital and temporal spikes and frequent bursts of generalized spike and wave complexes or generalized polyspike and wave complexes were recorded by EEG. Giant SEP and long-latency cortical reflex can be detected.. Irregular, arrhythmic or semi-rhythmic, high-frequency myoclonic jerks. Burst of 50-msec, irregular tremor with frequencie3s around 9-10 Hz. Cortiocomuscular coherence at 17 Hz. | Cortical myoclonus | Cortical myoclonus | Cortical myoclonus and myoclonic epilepsy |
| RPS6KA3 | Stimulus-induced negative myoclonus | N.A. | EEGs, including an EEG during a provoked drop episode, showed no epileptic abnormalities. SEP studies showed no giant potentials. | Non stated | Unknown | Unknown |
| SACS | Multifocal myoclonus. Spontaneous and movement-activated myoclonus. | N.A. | Diffuse spike and waves, giant SEP, enhanced long-loop reflexes. Photosensitivity. | Cortical myoclonus (Nascimento et al. 2016) | Unknown | Cortical myoclonus |
| SCARB2 | Action, spontaneous, stimulus-induced myoclonus, present in extremities | N.A. | Positive cortical spike back-averaging. Burst duration <50ms | Cortical myoclonus / Cortical tremor (Dibbens et al. 2016) | Cortical myoclonus | Cortical myoclonus |
| SCN1A & GABRA1 & SCN1B & PCDH19 | Interictal myoclonus, multifocal action myoclonus, spontaneous isolated myoclonic jerks. | Generalized myoclonic seizures, drop attacks. | Interictal EEG features include generalized, focal, and multifocal abnormalities and a marked photosensitivity in up to 40% of cases. During fragmented and segmental myoclonus, EEG shows diffuse slow waves intermixed with focal and diffuse spikes. EMG bursts in beta frequency during active movements, brief ranging from 24-48 ms. Giant SEP. Presence of cortico-muscular coherence. | Myoclonic epilepsy & Cortical myoclonus | Cortical myoclonus | Myoclonic epilepsy & cortical myoclonus |
| SERPINI1 | N.A. | Myoclonic seizures, negative myoclonic seizures | Bursts of generalized spike waves. Photosensitivity was not present. | None stated | Unknown | Myoclonic epilepsy |
| SGCE | Truncal and upper limb myoclonus | N.A. | Irregular bursts ranging from 30.1-750.6 ms. EEG back-averaging did not reveal cortical spikes or slow waves time-locked to muscle jerks | Subcortical myoclonus (Li et al. 2008) | Subcortical myoclonus | Subcortical myoclonus |
| SLC2A1 | N.A. | Myoclonic, myoclonic-astatic epilepsy | EEG shows generalized spike- or polyspike-wave activity above 2.5 Hz. | Myoclonic epilepsy | NA | Myoclonic epilepsy |
| SLC6A1 | N.A. | Drop attacks. Myoclonic-atonic seizures, characterized by abrupt cortical myoclonus or myoclonias followed by sudden loss of muscle control, often leading to a drop of fall. (Palmer et al. 2016) | All individuals had generalized spike-waves >2.5 Hz on their EEGs, and photo- paroxysmal response. | Myoclonic epilepsy | N.A. | Myoclonic epilepsy |
| SYNGAP1 | N.A. | Drop attacks, massive myoclonic jerks, atonic seizures, myoclonic absence or absences. | Ictal or interictal bursts of spikes, spike waves or slow waves that were either generalized or generalized with a posterior predominance. Paroxysmal anomalies were localized to central regions. Photosensitivity, fixation-off sensitivity were present. | Myoclonic epilepsy | N.A. | Myoclonic epilepsy |
| TBC1D24 | Upper extremity myoclonus, eyelids, face, lips, abdomen and limbs. Myoclonic episodes ranged from short, spontaneously resolving events. Preserved awareness with most episodes. | Myoclonic epilepsy, segmental or generalized, with initially no loss of consciousness but sometimes evolving into tonic-clonic seizures | Myoclonias of approximately 80-110s duration in all EMG channels, without any evidence of spread from one to another. A few myoclonias were more hypersynchronous, approximately 40-60ms in duration. Back-averaging did not reveal any preceding EEG change. SEP was normal | Myoclonic epilepsy. Myoclonus not further specified | Unknown | Cortical myoclonus & myoclonic epilepsy |
| UBE3A | Non-epileptical myoclonus. Lasting from several seconds to several minutes with the exception of one individual reporting near-constant myoclonus. In hands, spreading to extremities, face. Consciousness preserved. | Myoclonic, myoclonic absence and myoclonic-tonic seizures. Myoclonic status could appear. Some daily or nearly daily events, some sporadic (monthly to yearly). | No EEG correlate of myoclonus. Generalized spike and wave activity on EEG. | Cortical myoclonus & myoclonic epilepsy | Unknown | Unknown & myoclonic epilepsy |
