# Supplementary material for: Nomenclature of Genetically Determined Myoclonus Syndromes: Recommendations of the International Parkinson and Movement Disorder Society Task Force
Source: Mov Disord. 2019 Oct 4;34(11):1602–13. doi: 10.1002/mds.27828 (PMC6899848; doi:10.1002/mds.27828)
Supplement: Supplementary file 2 — Supplementary table 3 Overview of genes presenting with myoclonus [file MDS-34-1602-s002.docx]

**Genes presenting with prominent myoclonus, validated by a second independent research group**

| **Information source*** | **Gene name** | **Phenotype with prominent myoclonus?** | **Phenotype with prominent myoclonus described by two independent research groups?** | **Proven pathogenic for the following phenotypes according to OMIM:** |
| --- | --- | --- | --- | --- |
| Zutt *et al*. 2015 | AMT | Yes, majority | Yes | Glycine encephalopathy |
| Newly identified | CARS2 | Yes, majority | Yes | Combined oxidative phosphorylation deficiency type 27 |
| Zutt *et al*. 2015 | CLN3 | Yes, majority | Yes | Neuronal ceroid lipofuscinosis 3 |
| Zutt *et al*. 2015 | CHD2 | Yes, majority | Yes | Childhood-onset epileptic encephalopathy |
| Zutt *et al*. 2015 | CLN5 | Yes, majority | Yes | Neuronal ceroid lipofuscinosis 5 |
| Zutt *et al*. 2015 | CLN6 | Yes, majority | Yes | Neuronal ceroid lipofuscinosis 6 / Kufs type adult-onset |
| Zutt *et al*. 2015 | CLN8 | Yes, majority | Yes | Neuronal ceroid lipofuscinosis 8 / Northern epilepsy variant |
| Zutt *et al*. 2015 | CSTB | Yes, majority | Yes | Progressive myoclonic epilepsy 1A (Unverricht Lundborg disease) |
| Newly identified | CTSA | Yes, majority | Yes | Galactosialidosis |
| Newly identified | CUX2 | Yes, majority | Yes | Early infantile epileptic encephalopathy |
| Zutt *et al*. 2015 | DNAJC5 | Yes, majority | Yes | Neuronal ceroid lipofuscinosis 4 (Parry type) |
| Zutt *et al*. 2015 | EPM2A | Yes, majority | Yes | Progressive myoclonic epilepsy 2A (lafora disease) |
| Newly identified | FOLR1 | Yes, majority | Yes | Neurodegeneration due to cerebral folate transport deficiency |
| Zutt *et al*. 2015 | GABRA1 | Yes, majority | Yes | Early infantile epileptic encephalopathy 19 |
| Zutt *et al*. 2015 | GLDC | Yes, majority | Yes | Glycine encephalopathy |
| Zutt *et al*. 2015 | GLRA1 | Yes, majority | Yes | Hyperekplexia 1 |
| Zutt *et al*. 2015 | GLRB | Yes, majority | Yes | Hyperekplexia 2 |
| Zutt *et al*. 2015 | GOSR2 | Yes, majority | Yes | Progressive myoclonic epilepsy 6 |
| Zutt *et al*. 2015 | KCNC1 | Yes, majority | Yes | No |
| Zutt *et al*. 2015 | KCTD17 | Yes, majority | Yes | No |
| Zutt *et al*. 2015 | KCTD7 | Yes, majority | Yes | Progressive myoclonic epilepsy 3 with or without intracellular inclusions |
| Zutt *et al*. 2015 | MTTF | Yes, majority | Yes | Myoclonic epilepsy associated with ragged-red fibers |
| Zutt *et al*. 2015 | MTTH | Yes, majority | Yes | Myoclonic epilepsy associated with ragged-red fibers |
| Zutt *et al*. 2015 | MTTK | Yes, majority | Yes | Myoclonic epilepsy associated with ragged-red fibers |
| Zutt *et al*. 2015 | MTTL1 | Yes, majority | Yes | Myoclonic epilepsy associated with ragged-red fibers |
| Zutt *et al*. 2015 | MTTS1 | Yes, majority | Yes | Myoclonic epilepsy associated with ragged-red fibers |
| Zutt *et al*. 2015 | MTTS2 | Yes, majority | Yes | Myoclonic epilepsy associated with ragged-red fibers |
| Newly identified | MTTW | Yes, majority | Yes | Myoclonic epilepsy associated with ragged-red fibers |
| Zutt *et al*. 2015 | NEU1 | Yes, majority | Yes | Sialidosis type I and II |
| Zutt *et al*. 2015 | NHLRC1 | Yes, majority | Yes | Progressive myoclonic epilepsy 2B (Lafora disease) |
| Zutt *et al*. 2015 | PIGA | Yes, majority | Yes | Multiple congenital anomalies-hypotonia-seizures syndrome 2; somatic paroxysmal nocturnal hemoglobinuria |
| Zutt *et al*. 2015 | POLG | Yes, majority | Yes | Mitochondrial DNA depletion syndrome 4A (Alpers type); Mitochondrial DNA depletion syndrome 4B (MNGIE type); Mitochondrial recessive ataxia syndrome (includes SANDO and SCAE); Progressive external ophthalmoplegia AD & AR |
| Zutt *et al*. 2015 | PRICKLE1 | Yes, majority | Yes | No |
| Newly identified | RAPGEF2 | Yes, majority | Yes | No |
| Newly identified | SAMD12 | Yes, majority | Yes | No |
| Zutt *et al*. 2015 | SCARB2 | Yes, majority | Yes | Progressive myoclonic epilpesy 4 with or without renal failure |
| Zutt *et al*. 2015 | SCN1A | Yes, majority | Yes | Generalized epilepsy with febrile seizures plus type 32; early infantile epileptic enceophalopathy (Dravet syndrome); familial febrile seizures; familial hemiplegic migraine 3 |
| Newly identified | SERPINI1 | Yes, majority | Yes | Familal encephalopathy with neuroserpin inclusion bodies |
| Zutt *et al*. 2015 | SGCE | Yes, majority | Yes | Myoclonic-dystonia type 11 |
| Newly identified | SLC6A1 | Yes, majority | Yes | Myoclonic-atonic epilepsy |
| Zutt *et al*. 2015 | SLC6A5 | Yes, majority | Yes | Hyperekplexia type 3 |
| Newly identified | SYNGAP1 | Yes, majority | Yes | No |
| Newly identified | TBC1D24 | Yes, majority | Yes | Deafness (AR86 and AD65), deafness onychodystrophy osteodystrophy mental retardation and seizures syndrome (DOORS syndrome); EIEE 16 |
| Zutt *et al*. 2015 | TPP1 | Yes, majority | Yes | Neuronal ceroid lipofuscinosis 2; spinocerebellar ataxia 7 |
| Zutt *et al*. 2015 | ADCY5 | Yes, minority | Yes | Familial dyskinesia with facial myokymia |
| Zutt *et al*. 2015 | ANO3 | Yes, minority | Yes | Dystonia 24 |
| Newly identified | APP | Yes, minority | Yes | Familial Alzheimer disease; cerebral amyloid angiopathy (Dutch, Italian, Iowa, Flemish, Arctic variants) |
| Zutt *et al*. 2015 | ASAH1 | Yes, minority | Yes | Farber lipogranulomatosis ; spinal muscular atrophy with progressive myoclonic epilepsy |
| Zutt *et al*. 2015 | ATM | Yes, minority | Yes | Ataxia-telangiectasia; lymphoma, B-cell non-Hodgkin, somatic; Lymphoma, mantle cell, somatic; T-cell prolymphocytic leukemia, somatic |
| Zutt *et al*. 2015 | ATN1 | Yes, minority | Yes | Dentotorubro-pallidoluysian atrophy |
| Newly identified | CSNK2B | Yes, minority | Yes | No |
| Newly identified | FARS2 | Yes, minority | Yes | Combined oxidative phosphorylation deficiency 14; spastic paraplegia type 77 |
| Zutt *et al*. 2015 | GBA | Yes, minority | Yes | Gaucher disease, perinatal lethal / type I / type II / type III / type IIIC |
| Zutt *et al*. 2015 | HTT | Yes, minority | Yes | Huntington disease |
| Newly identified | KIF5A | Yes, minority | Yes | Hereditary spastic paraplegia |
| Zutt *et al*. 2015 | mUDPC7 | Yes, minority | Yes | Silver-Russell syndrome |
| Zutt *et al*. 2015 | NKX2-1 | Yes, minority | Yes | Brain-lung-thyroid syndrom; benign hereditary chorea; |
| Zutt *et al*. 2015 | NPC1 | Yes, minority | Yes | Niemann-pick disease, type C1 / type D |
| Zutt *et al*. 2015 | PCDH19 | Yes, minority | Yes | EIEE type 9 |
| Zutt *et al*. 2015 | PRKCG | Yes, minority | Yes | Spinocerebellar ataxia 14 |
| Zutt *et al*. 2015 | PRNP | Yes, minority | Yes | Cerebral amyoloid angiopathy, PRNP-related ; Creutzfeldt-Jakob disease; Gerstmann-Straussler disease; Huntington disease-like 1; insomnia fatal familial; prion disease with protracted course |
| Zutt *et al*. 2015 | PSEN1 | Yes, minority | Yes | Alzheimer disease type 3 / type 3 with spastic paraparesis and apraxia / type 3 with spastic paraparesis and unusual plagues; dilated cardiomyopathy 1U; frontotemporal dementia; Pick disease |
| Zutt *et al*. 2015 | RPS6KA3 | Yes, minority | Yes | Coffin-Lowry syndrome; x-linked mental retardation type 19 |
| Newly identified | SACS | Yes, minority | Yes | Charlevoix-Sanguenay spastic ataxia |
| Zutt *et al*. 2015 | SCN1B | Yes, minority | Yes | Generalized epilepsy with febrile seizures plus type 1; EIEE type 52; familial atrial fibrillation type 13; brugada syndrome type 5; nonspecific cardiac conduction defect |
| Zutt *et al*. 2015 | SLC2A1 | Yes, minority | Yes | Glucose transport type 1 deficiency syndrome (Glut1-DS), paroxysmal exertion-induced dyskinesia (PED), early onset absence epilepsy (EOAE), childhood absence epilepsy, episodic choreoathetosis and spasticity (CSE) |
| Zutt *et al*. 2015 | UBE3A | Yes, minority | Yes | Angelman syndrome |

**Genes presenting with prominent myoclonus; however, phenotype not confirmed by a second independent research group**

| **Information source*** | **Gene name** | **Phenotype with prominent myoclonus?** | **Phenotype with prominent myoclonus described by two independent research groups?** | **Proven pathogenic for the following phenotypes according to OMIM:** |
| --- | --- | --- | --- | --- |
| Newly identified | ADGRV1 | Yes, myoclonic epilepsy (Myers et al. 2018) | No | Usher syndrome type 2c |
| Newly identified | AIFM1 | Yes, minority (Heimer et al. 2018) | No | Combined oxidative phosphorylation deficiency 6; Chowchock syndrome; X-linked deafness type 5 |
| Zutt *et al*. 2015 | ARHGEF9 | Yes, hyperekplexia in minority (Harvey et al. 2004) | No | Epilepsy with intellectual disability (Wang et al. 2018) |
| Zutt *et al*. 2015 | ATP13A2 | Yes, progressive myoclonus epilepsy in minority (Bras et al. 2012) | No | Generalized dystonia with non-prominent myoclonus (Schneider et al. 2010); Kufor-Rakeb syndrome; spastic paraplega 78 |
| Zutt *et al*. 2015 | CACNA1A | Yes, progressive myoclonus epilepsy in minority (LV et al. 2017) and neonatal myoclonic epilepsy (Epi4K consortium 2013 & 2016) (two separate phenotypes) | No | Familial hemiplegic migraine type 1, SCA 6 and EA2 |
| Zutt *et al*. 2015 | CERS1 | Yes, progressive myoclonus epilepsy in majority (Ferlazzo et al. 2016) | No | No |
| Newly identified | CPLX1 | Yes, myoclonic epilepsy in majority (Redler et al. 2017) | No | Early infantile epileptic encephalopathy 63 |
| Zutt *et al*. 2015 | CYP27A1 | Yes, myoclonus-dystonia in minority (Lagarde et al. 2012) | No | Cerebrotendinous xanthomatosis with non-prominent myoclonus (Lagarde et al. 2012) |
| Zutt *et al*. 2015 | EIF2B5 | Yes, progressive myoclonus epilepsy in minority (Jansen et al. 2008) | No | Leukoencephalopathy with vanishing white matter, non-dominent myoclonus has been described (Sharma et al. 2011) |
| Newly identified | GABRB2 | Yes, myoclonic epilepsy | No | Infantile or early childhood epileptic encephalopathy 2 |
| Newly identified | GABRB3 | Yes, myoclonic epilepsy in minority (Le et al. 2017) | No | EIEE type 43 (including unconfirmed Doose syndrome) |
| Additional literature | GPHN | Yes, hyperekplexia in majority (Rees et al. 2003) | No | Molybdenum cofactory deficiency C |
| Newly identified | HCN1 | Yes, myoclonic epilepsy in minority (Nava et al. 2014) | No | EIEE type 24 |
| Zutt *et al*. 2015 | KCND3 | Yes, myoclonus-dystonia in minority (Kurihara et al. 2018) | No | Yes, Brugada syndrome 9 and spinocrerebellar ataxia 19 with non-dominant myoclonus |
| Newly identified | KCNQ2 | Yes, myoclonic epilepsy in minority (Kojima et al. 2018) | No | EIEE type 7; benign neonatal seizures type 1 |
| Newly identified | LAMC3 | Yes, myoclonic epilepsy in minority (Afawi et al. 2016) | No | Cortical malformations, occipital |
| Newly identified | LMNB2 | Yes, progressive myoclonus epilepsy in majority(Damiano et al. 2015) | No | No |
| Additional literature | MRE11 | Yes, progressive myoclonus epilepsy in minority (Miyamoto et al. 2014) | No | Ataxia-telangiectasia-like disorder |
| Newly identified | PIGL | Yes, myoclonic epilepsy in minority (Mogami et al. 2018) | No | CHIME syndrome |
| Newly identified | RARS2 | Yes, myoclonic epilepsy in minority (Mathew et al. 2018) | No | Pontocerebellar hypoplasia type 6 in which no myoclonus/myoclonic epilepsy has ever been reported |
| Zutt *et al*. 2015 | RELN | Yes, myoclonus-dystonia in majority (Groen et al. 2015) | No | Lissencephaly 2 (Norman-Roberts type) |
| Zutt *et al*. 2015 | SCN2A | Yes, myoclonic epilepsy in minority (Steel et al. 2017) | No | Early infantile, epileptic encephalopathy 11; benign familial infantile seizures 3 |
| Newly identified | SLC25A22 | Yes, myoclonic epilepsy | No | Early infantile epileptic encephalopathy 3 |
| Additional literature | SLC25A46 | Yes, progressive myoclonus ataxia in minority (Charlesworth et al. 2016) | No | Hereditary motor and sensory neuropathy type VIB |
| Newly identified | SMARCA2 | Yes, myoclonus epilepsy in minority (Tang et al. 2017) | No | Nicolaides-Baraitser syndrome |
| Zutt *et al*. 2015 | SNCA | Yes, myoclonus in minority (Puschmann et al. 2008) | No | Lewy body dementia; Parkinsons disease 1 & 4 |
| Zutt *et al*. 2015 | STXBP1 | Yes, myoclonic epilepsy in minority (Dravet syndrome) | No | EIEE type 4 |
| Zutt *et al*. 2015 | TH | Yes, myoclonus-dystonia in minority (Stamelou et al. 2012) | No | Segawa syndrome |
| Newly identified | TNRC6A | Yes, familial cortical myoclonic tremor with epilepsy (Ishiura et al. 2018 | No |  |
| Newly identified | TUBB2B | Yes, myoclonus-dystonia in minority (Geiger et al. 2017) | No | Cortical dysplasia with delayed psychomotor development and seizures |
| Newly identified | UBA5 | Yes, myoclonic epilepsy (Mignon-Ravix et al. 2018) | No | EIEE type 42 with non-dominant myoclonic jerks |

**Genes presenting with prominent myoclonus; however, with questionable pathogenicity**

| **Information source*** | **Gene name** | **Phenotype with prominent myoclonus?** | **Phenotype with prominent myoclonus described by two independent research groups?** | **Proven pathogenic for the following phenotypes according to OMIM:** |
| --- | --- | --- | --- | --- |
| Newly identified | BSCL2 | Yes, myoclonic epilepsy in minority (Opri et al. 2016 & Guillén-Navarro et al. 2013) | Questionable pathogenicity as mutation in BSCL2 was only hypothesized in two of three patients for gene molecular analysis was not performed (Opri et al. 2016); Guillén-Navarro et al (2013) described 6 patients; however, no DNA was available in the ones with predominant myoclonus | Progressive encephalopathy with or without lipodystrophy; congenital generalized lipodystrophy type 2; hereditary distal motor neuropathy type VA; Silver spastic paraplegia syndrome |
| Zutt *et al*. 2015 | GABRG2 | Yes, myoclonic epilepsy in minority (Harkin et al. 2002) | Reported by a second independent research group; however epilepsy inheritance questionable (Harkin et al. 2002) | Generalized epilepsy with febrile seizures plus type 3; familial febrile seizures type 8 |
| Zutt *et al*. 2015 | CACNA1B | Yes, myoclonus-dystonia in majority (Groen et al. 2015) | No | No |
| Newly identified | ACMSD | Yes, FCMTE | Authors believe TTTCA and TTTTA pentanucleotide repeates of introns to be causative of FCMTE irrespective of the genes in which they are located (Ishiura et al. 2018) | No |
| Newly identified | ADRA2B | Yes, FCMTE |  | No |
| Zutt *et al*. 2015 | CNTN2 | Yes, FCMTE |  | No |
| Newly identified | NOL3 | Yes, FCMTE |  | No |
| Newly identified | PLA2G6 | Yes, FCMTE |  | Infantile neuroaxonal dystrophy 1; NBIA 2B; parkinsons disease 14, autosomal recessive |
| Newly identified | SLC30A8 | Yes, FCMTE |  | No |
| Newly identified | UBR5 | Yes, FCMTE |  | No |
| Zutt *et al*. 2015 | BRD2 | Yes, JME | JME does not fit the fully penetrant monogenic disease model and pathogenicity is therefore hard to determine. Recently, EFHC1 (most studied gene of JME) has been stated as definitely implicated by NHGRI and ACMG guidelines (Bailey *et al*. 2017). However pathogenicity remains questionable. | No |
| Zutt *et al*. 2015 | CACNB4/EJM6 | Yes, JME |  | Episodic ataxia, type 5 |
| Zutt *et al*. 2015 | CASR | Yes, JME |  | Hyperparathyroidism, neonatal; hypocalcemia, AD; hypocalciuric hypercalcemia, type I |
| Zutt *et al*. 2015 | CHRNA7 | Yes, JME |  | No |
| Zutt *et al*. 2015 | CLCN2 | Yes, JME |  | Hyperaldosteronism, familial, type II; leukoencephalopathy with ataxia |
| Zutt *et al*. 2015 | EFHC1 | Yes, JME |  | No |
| Zutt *et al*. 2015 | EFHC2 | Yes, JME |  | No |
| Zutt *et al*. 2015 | GABRD | Yes, JME |  | No |
| Zutt *et al*. 2015 | GJD2 | Yes, JME |  | No |
| Newly identified | GRM4 | Yes, JME |  | No |
| Newly identified | ICK | Yes, JME |  | Endocrine-cerebro-osteodysplasia |
| Zutt *et al*. 2015 | ME2 | Yes, JME |  | No |
| Newly identified | TOP3B | Yes, JME |  | No |

**Genes presenting with myoclonus in some cases; however, no prominent myoclonus is described.**

| **Information source*** | **Gene name** | **Phenotype with prominent myoclonus?** | **Phenotype with prominent myoclonus described by two independent research groups?** | **Proven pathogenic for the following phenotypes according to OMIM:** |
| --- | --- | --- | --- | --- |
| Newly identified | AAAS | No | N.A. | Allgrove syndrome |
| Newly identified | ALDH7A1 | No | N.A. | Pyridoxine-dependent epilepsy |
| Newly identified | ATP1A3 | No | N.A. | Alternating hemiplegia of childhood 2, CAPOS syndrome and dystonia type 12 |
| Zutt *et al*. 2015 | ATP7B | No | N.A. | Wilson disease |
| Zutt *et al*. 2015 | ATXN1 | No | N.A. | Spinocerebellar ataxia 1 |
| Zutt *et al*. 2015 | ATXN2 | No | N.A. | Spinocerebellar ataxia 2 |
| Zutt *et al*. 2015 | ATXN3 | No | N.A. | Machado-Joseph disease |
| Zutt *et al*. 2015 | ATXN7 | No | N.A. | Spinocerebellar ataxia 7 |
| Zutt *et al*. 2015 | ATXN8 | No | N.A. | Spinocerebellar ataxia 8 |
| Zutt *et al*. 2015 | ATXN8OS | No | N.A. | Spinocerebellar ataxia 8 |
| Newly identified | BOLA3 | No | N.A. | Multiple mitochondrial dysfunctions syndrome 2 with hyperglycinemia |
| Newly identified | BRAT1 | No | N.A. | Neurodevelopmental disorder with cerebellar atrophy and with or without seizures; rigidity and multifocal seizure syndrome, lethal neonatal |
| Newly identified | CPA6 | No | N.A. | Familial temporal lobe epilepsy type 5; familial febrile seizures type 11 |
| Newly identified | DNM1L | No | N.A. | Optic atrophy 5 |
| Zutt *et al*. 2015 | FXN | No | N.A. | Friedreich ataxia |
| Zutt *et al*. 2015 | GALC | No | N.A. | Krabbe disease |
| Zutt *et al*. 2015 | GCSH | No | N.A. | No |
| Zutt *et al*. 2015 | GFAP | No | N.A. | Alexander disease |
| Zutt *et al*. 2015 | GRN | No | N.A. | Primary progressive aphasia; neuronal ceroid lipofuscinosis 11; frontotemporal lobar degeneration with ubiquitin-positive inclusions |
| Zutt *et al*. 2015 | HEXA | No | N.A. | GM2-gangliosidosis; Tay-Sachs disease |
| Zutt *et al*. 2015 | HEXB | No | N.A. | Sandhoff disease, infantile, juvenile, and adult forms |
| Zutt *et al*. 2015 | JAK3 | No | N.A. | SCID, autosomal recessive, T-negative/B-positive type |
| Newly identified | KMT2B | No | N.A. | Childhood-onset dystonia type 28 (Kawarai et al. 2018) |
| Zutt *et al*. 2015 | MAPT | No | N.A. | Frontotemporal dementie, with or without parkinsonism; Pick disease; progressive supranuclear palsy (atypical) |
| Zutt *et al*. 2015 | MECP2 | No | N.A. | Neonatal severe encephalopathy; X-linked syndromic mental retardation, Lubs type; X-linked syndromic mental retardation type 13; Rett syndrome, Rett syndrome, atypical; Rett syndrome, preserved speech variant |
| Zutt *et al*. 2015 | MFSD8 | No | N.A. | Neuronal ceroid lipofuscinosis 7; macular dystrophy with central cone involvement |
| Zutt *et al*. 2015 | MTATP6 | No | N.A. | No |
| Zutt *et al*. 2015 | NEXMIF | No | N.A. | X-linked, mental retardation type98 |
| Zutt *et al*. 2015 | NPC2 | No | N.A. | Niemann-pick disease, type C2 |
| Zutt *et al*. 2015 | PANK2 | No | N.A. | HARP syndrome; neurodegeneration with brain iron accumulation type 1 |
| Zutt *et al*. 2015 | PPT1 | No | N.A. | Neuronal ceroid lipofuscinosis 1 |
| Zutt *et al*. 2015 | PSAP | No | N.A. | Combined SAP deficiency; Guacher disease, atypical; Krabbe disease, atypical; metachromatic leukodystrophy due to SAP-b deficiency |
| Newly identified | PSEN2 | No | N.A. | Alzheimer disease type 4; Cardiomyopathy, dilated 1V |
| Zutt *et al*. 2015 | RNASEH2B | No | N.A. | Aicardi-Goutieres syndrome 2 |
| Zutt *et al*. 2015 | RNASEH2C | No | N.A. | Aicardi-Goutieres syndrome 3 |
| Zutt *et al*. 2015 | SAMHD1 | No | N.A. | Aicardi-Goutieres syndrome 5 |
| Zutt *et al*. 2015 | SCN8A | No | N.A. | EIEE type 13; benign familial infantile seizures type 5 |
| Zutt *et al*. 2015 | TBP | No | N.A. | Spinocerebellar ataxia 17 |
| Zutt *et al*. 2015 | TREX1 | No | N.A. | Aicardi-Goutieres syndrome 1, dominant and recessive; Chilblain lupus; Vasculopathy, retinal, with cerebral leukodystrophy |
| Zutt *et al*. 2015 | ATP7A | No | N.A. | Menkes disease; occipital horn syndrome; spinal muscular atrophy, X-linked 3 |
| Zutt *et al*. 2015 | CDKL5 | No | N.A. | Early infantile epileptic encephalopathy 2 |
| Newly identified | KCNA2 | No | N.A. | Early infantile epileptic encephalopathy 32 |
| Newly identified | KIF1A | No | N.A. | Mental retardation type 9; hereditary sensory neuropathy type IIC; spastic paraplegia type 30 |
| Zutt *et al*. 2015 | SCN9A | No | N.A. | Generalized epilepsy with febrile seizures plus type 7; primary erythermalgia; familial febrile seizures; HSAN2D; congenital insensitivity to pain; paroxysmal extreme pain disorder; small fiber neuropathy |
| Newly identified | SIK1 | No | N.A. | Early infantile epileptic encephalopathy 30 |
